# Otodental syndrome

**DOI:** 10.1186/1750-1172-1-5

**Published:** 2006-03-21

**Authors:** Agnès Bloch-Zupan, Jane R Goodman

**Affiliations:** 1 Faculté de Chirurgie Dentaire de Strasbourg, Université Louis Pasteur Hôpital Civil, 1 place de l'Hôpital 67000 Strasbourg, France; 2 Eastman Dental Institute, University College London, 256 Gray's Inn Road, London, WC1X 8LD, UK

## Abstract

The otodental syndrome also named otodental dysplasia, is characterised by a striking dental phenotype known as globodontia, associated with sensorineural high frequency hearing loss and eye coloboma. Globodontia occurs in both primary and permanent dentition, affecting canine and molar teeth (*i.e*. enlarged bulbous malformed posterior teeth with almost no discernable cusps or grooves). The condition appears to be inherited in an autosomal dominant mode, although sporadic cases have been reported. It is a rare disease, a few families have been described in the literature. In the British family, the locus for oculo-oto-dental syndrome was mapped to 20q13.1 within a 12-cM critical chromosomal region. Dental management is complex, interdisciplinary and will include regular follow up, scheduled teeth extraction and orthodontic treatment. Hearing checks and, if necessary, hearing aids are mandatory, as well as eye examination and *ad hoc *treatment if necessary.

## Disease name and synonyms

The Otodental syndrome has been described under various names:

• Otodental dysplasia [1,2];

• Familial otodentodysplasia [3];

• Globodontia; the term globodontia refers to the enlarged bulbous fused malformed posterior teeth with almost no discernable cusps or grooves [4-6];

• In some families, an associated ocular phenotype was recognized [7] and it was named as the Oculo-Oto-Dental syndrome (OOD) [8].

## Epidemiology

The condition has been described in at least 9 families. The first case of otodental syndrome was described in Hungary in a mother and her son by Denes and Csiba, 1969 [9]. So far a British kindred [7,8], a girl of Irish extraction [5], a family from Brazil [10,11], a Chinese boy [1], a family of Polish extraction [4,6], an Austrian family [12], a kindred of Italian extraction followed through six generations [2,3,13,14], and a Belgian family [15] have been described in the literature.

## Definition/Diagnosis criteria

The otodental syndrome, also named otodental dysplasia, is characterised by a striking dental phenotype known as globodontia, associated with sensorineural high frequency hearing loss and eye coloboma.

The dental phenotype is *per se *diagnostic. It consists mainly of enlarged canine and molar fused teeth (globodontia, displaying globe-shaped crowns) both in the primary and in the permanent dentition. The incisors are not affected. In a few cases described in the literature the condition was discovered in young children (around 3 years of age) [1,11] consulting for delayed eruption of their posterior teeth.

Sensorineural high frequency hearing loss and coloboma was reported in the British family only [7,8].

Some authors have reported dysmorphic facial features. Witkop *et al*., 1976 [6] described patients with a long facies, anteverted nostrils, a long philtrum and a full cheek appearance. The patients case 2 and 3 described by Vieira *et al*., 2002 [8] had also a long face, full cheek and a strong mentalis muscle activity in electromyography. The patient's father and son examined by Jorgenson *et al*., 1975 [14] had symmetric and normognathic facies. Several small deeply pigmented lesions nevi existed over their faces and scalp. Their ears were protuberant.

Constitutional short stature was diagnosed in a patient followed by Levin *et al*., 1975 [16]. However, other individuals of the same family presenting otodental dysplasia were of normal stature.

## Differential diagnosis

The association of sensorineural hearing loss and dental anomalies can be found in other syndromes:

• autosomal recessive sensorineural hearing impairment, dizziness and hypodontia [17];

• bilateral sensorineural hearing loss and multiple anterior dens invaginatus [18];

• Kantaputra and Gorlin, 1992 [19] described molarization and premolarization of anterior teeth in a double dens invaginatus in an affected female who had developmental delay and congenital progressive sensorineural hearing loss. In addition, multituberculated mandibular incisors, canines, and first premolar were observed. None of these entities however display the striking dental phenotype detailed below [19].

## Aetiology

The condition appears to be inherited on an autosomal dominant basis. In the British family described by Vieira *et al*., 2002 [8], the locus for *OOD *was mapped to 20q13.1 within a 12-cM critical chromosomal region.

## Clinical description

### Sensorineural high frequency hearing loss

Sensorineural hearing loss of about 65 dB is found at all frequencies but is more pronounced at about 1000 Hz. It usually plateaus by the fourth decade [13]. The age of onset varies from early childhood to middle age [13,14,16]. Hearing loss is progressive and bilateral [13]. It was described as starting in infancy and progressing to a plateau by approximately 35 years of age by Vieira *et al*., 2002 [8]. Speech defects were minor.

Frequent ear abscesses were noted in one patient [8].

Differential diagnostic audiometric test suggested a cochlear site of lesion [13].

### Eye phenotype

Eye phenotype was described by Vieira *et al*., 2002 [8]. Abnormalities ranged from transillumination defects in the inferior iris, due to iris pigment epithelium defects, to severe chorioretinal coloboma. Other ocular signs were microcornea, microphthalmos, lens opacity and lens coloboma. Marked asymmetry in eye signs was seen in some individuals.

### Oral/Dental anomalies

The gingiva was described as inflamed and enlarged in a patient [14]. Gingival hyperplasia was a common clinical finding around erupting teeth [8].

The oral/dental anomalies affect both the primary and the permanent dentition and could be classified as anomalies of eruption, number, size, shape and structure.

### Eruption

There was a significant delay in eruption of the primary and permanent dentition [8,16], especially in the lateral sectors [13]. If the premolars were absent, the deciduous molars were retained. The primary teeth might exfoliate later than average [13].

### Tooth number

Missing teeth, especially premolars, were reported [16]. The premolars which were present might be smaller but of normal morphology. Numerous supernumerary microdont teeth were also described by Chen *et al*., 1988 [1] and Winter, 1983 [7].

### Shape and size

Large bulbous canines and molars crowns can summarize the clinical intraoral findings.

Permanent molars are malformed with fusion of cusps. The crowns of the canines and posterior teeth are enlarged, bulbous and malformed with multiple prominent lobules. The deciduous dentition is more severely involved. The relation between cusps and the major groove is eliminated hence the use of the term *globodontia *[18]. Cook *et al*., 1981 [13] described the specific shape of the crowns as an unusual number of well-developed cusps and lack of developmental grooves and fossa on the cusps was noted. Jorgenson *et al*., 1975 [14] noted large and bulbous primary canine crowns but normal successional permanent canines.

The canines have been described as large with a marked bulbous cingulum [11]. Based on the morphology, it might be difficult to distinguish deciduous from permanent teeth [20].

The average size premolars had convex occlusal surfaces with no developmental grooves or fossae [13]. In the cases described by Toledo *et al*., 1971 [20] premolars were also described as presenting bulbous crowns and rounded cusps. Sedano *et al*., 2001 [20] stated that in some patients the large abnormal molars could be the product of fusion of premolars and molar tooth germs. The crowns of the incisors were normal in size and shape.

### Enamel structure

An enamel defect (hypoplasia) was frequently found on the buccal surface of canines [18].

The teeth might be prone to decay [13]. The enamel appeared hypoplastic, pitted (yellow teeth) [8]. Vieira *et al*., 2002 insisted that the incisors were not normal, demonstrating also the yellowing and pitting enamel defects [8].

### Roots

The molars displayed taurodontism (an inverted crown-body/root ratio). The roots were short and tapered and some were taurodont in configuration [14]. The deciduous molars appeared to have two separate pulp chambers, giving the impression that either fusion or gemination had occurred [16]. The pulp chambers of the molars appeared duplicated [11]. Large calcifications were present in the pulp chambers and root canals of deciduous teeth. Pulp chambers of the posterior teeth displayed a thistle-tube configuration [14].

Vieira *et al*., 2002 described also the enlarged pulp chambers, the pulp stones and the abnormal root defects [8].

### Occlusion

Various malocclusions were described in the family members examined by Vieira *et al*. [8]. Case 1 demonstrated a posterior bilateral crossbite. The mandibular arch was "U" shaped and the maxillary arch was "V" shaped and constricted with a deep palate. Case 2 and 3 presented a malocclusion with anterior open bite and maxillary lateral incisors palatal to the central incisors.

The boy presented by Chen *et al*., 1988 [1] had an occlusion compatible with an Angle's Class III molar relationship.

### Tumor

Odontomas, the most common type of odontogenic tumors, were reported by Beck-Mannagetta *et al*., 1984 [12] in the posterior maxilla and mandible of a father and his daughter affected by the otodental syndrome.

## Histopathology

The histological findings described by Beck-Mannagetta *et al*., 1984 and Toledo *et al*., 1971 [12,20] showed no evidence of structural anomalies in the enamel, dentin or pulp.

In the area of hypoplastic enamel, slightly reduced enamel thickness and alterations existed with prominent enamel rods, irregular incremental lines and rod sheath area containing voids, defects very similar to those observed in hypomaturation enamel defects. The amelodentinal junction in these areas was displaced towards the surface of the tooth and the subjacent underlying dentin had scanty irregular tubules [6].

## Genetic counselling

Genetic counselling is important. Inheritance is clearly autosomal dominant with complete [8] to variable penetrance [11] according to the authors, and a variable expressivity. However, other genetic or environmental effects may possibly influence disease severity and could explain the marked eye phenotype asymmetry in individual patients [8].

## Management including treatment

Dental management is complex, interdisciplinary and will include regular follow up, scheduled tooth extraction and eventually orthodontic treatment. Hearing check and, if necessary, hearing aids are mandatory; as well as eye examination and *ad hoc *treatment if necessary.

## Unresolved questions

The identification of the gene involved in this disease is of importance in our understanding of the development of various tissues and organs (teeth, ear, eye...).
